# Immunomodulatory and Antioxidant Potential of Biogenic Functionalized Polymeric Nutmeg Oil/Polyurethane/ZnO Bionanocomposite

**DOI:** 10.3390/pharmaceutics13122197

**Published:** 2021-12-19

**Authors:** Musarat Amina, Nawal M. Al Musayeib, Nawal A. Alarfaj, Maha F. El-Tohamy, Gadah A. Al-Hamoud, Hanan M. Al-yousef

**Affiliations:** 1 Department of Pharmacognosy, Pharmacy College King Saud University, Riyadh 11451, Saudi Arabia; nalmusayeib@ksu.edu.sa (N.M.A.M.); galhamoud@ksu.edu.sa (G.A.A.-H.); halyousef@ksu.edu.sa (H.M.A.-y.); 2 Department of Chemistry, College of Science, King Saud University, P.O. Box 22452, Riyadh 11451, Saudi Arabia; nalarfaj@ksu.edu.sa (N.A.A.); moraby@ksu.edu.sa (M.F.E.-T.)

**Keywords:** *Myristica fragrans* seeds, polyurethane, bionanocomposite, immunomodulatory, antimicrobial

## Abstract

The current study is focused on the biosynthesis of nutmeg oil/ polyurethane/ZnONPs bionanocomposite film for immunomodulatory and antioxidant activities. The fabricated film was prepared by using naturally extracted nutmeg oil functionalized with ZnONPs in the presence of polyutherane (PU) medium. The bionanocomposite film was obtained by incorporating dropwise 10 % (*w*/*v*) of nutmeg oil to the PU solution/ZnONPs blend. The active constituents of nutmeg oil were determined by gas chromatography coupled with mass spectrometry (GC-MS). The morphological characteristics of the resulting bionanocomposite film were confirmed using various microscopic and spectroscopic methods. Immunomodulatory potential of bionanocomposite was evaluated for RAW 264.7 macrophages. The results exhibited an excellent reduction in inflammatory cytokines (IL-6, IL-10, and TNFα) secretions after the treatment with bionanocomposite. The bionanocomposite exerted the highest inhibitory effects on certain cell signaling constituents that influence the initiation of expression of proinflammatory cytokines. The bionanocomposite was also tested for DPPH and ABTS free radicals scavenging assays and showed excellent antioxidant potential with IC_50_ values (0.28 ± 0.22 and 0.49 ± 0.36), respectively. The outcomes suggested promising immunomodulatory and antioxidant potentials for the biogenic synthesized nutmeg oil/PU/ZnONPs polymeric bionanocomposite.

## 1. Introduction

Nanoparticles have been extensively studied over the last decade due to their unique features such as optical, electrical, magnetic, mechanical, thermal, catalytic, dielectric, and biological properties [1]. They are considered as the main building blocks of the next generation technology with tremendous applications in various industrial and biomedical sectors [2]. In particular, metal and metal oxide nanoparticles are gaining much attention due to their distinctive electronic, optical, and magnetic characteristics [3]. The oxides of transition metals are crucial semiconductors that showed useful properties in electronics, solar energy transformation, gas sensors, magnetic storage media, and catalysis [4,5,6]. Although various chemical and physical techniques have been extensively applied to synthesize metal oxide nanostructures including, hydrothermal, microemulsion, sonochemical, arc-submerged, flame-based aerosol, and solid-state methods [7,8,9,10,11]. However, the use of toxic chemicals and their stability are the subjects of supreme concern. The utilization of hazardous chemicals on nanoparticles’ surface and nonpolar organic solvent in the synthesis method restricts their applications in clinical and medical fields. Hence, the development of non-toxic, clean, eco-friendly, and biocompatible procedures for the synthesis of nanoparticles deserve merit. The recent interest has shifted towards bioprocessing and the green chemistry approach. These methodologies are concerned with the use of cost-effective, biocompatible, and eco-friendly reducing agents for the preparation of nanomaterials. Green synthesis of nanomaterials has captured enormous attention due to their environmentally-benign, compatibility with living creatures, various biomedical properties, and efficient ability to remove waste products [12,13,14,15]. The literature survey revealed that the synthesis of nanomaterials using natural products (plants, sponges, cyanobacteria, fungi, and algae) has been explored. The combination of plant-derived bioactive molecules with synthetic biomaterials for the preparation of nanomaterials has been utilized for various biological purposes [16,17]. Oil-based polymeric bionanocomposite films have displayed great advantages and extensive prospective use in many applications including pharmaceutical, biomedical, and packaging devices [18,19,20]. Furthermore, nanomaterials in a combination with metal and metal oxides exhibited various viable and significant biological properties [21]. Considering the health benefits of essential oils (EOs) to human beings from ages, the metal/metal oxide nanostructures in combination with natural oils has unfolded new approaches to design biomaterials with several biomedical applications [22].

Nutmeg (*Myristica fragrans*) is the most popular commercial species of the genus, indigenous to the Banda Island of Indonesia and its therapeutics applications are well recorded in Arabian physicians in the last seventh century of C.E. (common era) [23]. It is widely used in food condiments, flavoring agent, perfumery, and medicine. The nutmeg exhibited various biological properties such as antispasmodic, analgesic, antiseptic, astringent, aphrodisiac, hemostatic, carminative, sedative, anti-inflammatory, insecticidal, and parasiticide [24]. Nutmeg seeds are rich source of essential oil, fatty acids, resins, wax, and other components. The essential oil gives nutmeg a distinctive fragrance and flavoring as a culinary ingredient [25]. The main constituents reported from nutmeg essential oil were terpenes and phenylpropanoids. Nutmeg essential oil has been widely reported as a disinfectant, antithrombotic, anti-inflammatory, anti-dysentery agent rheumatism, narcotic, and antimicrobial properties [25,26].

Recently, the application of nanoscience for the preparation of modern metal/metal oxide nanostructures with enhanced surface area, efficient absorptivity and advanced catalytic potential has affected both science, and industry [27]. The formation of hybrid nanocomposites open up promising aspiration for the solution to various health issues in humans [28]. Among various metal oxide nanostructures, zinc oxide nanoparticles (ZnONPs) have been used for several biological applications due to their excellent stability and coherent biocompatibility [29]. ZnONPs exhibited remarkable potential in the biomedical fields including, antibacterial, antifungal, anticancer, drug delivery systems, and osteogenesis because of their efficient penetration capability and interaction with cells and tissues [30]. Moreover, the combination of ZnONPs with natural products/essential oils can enhance the biological applications against various disorders [31]. However, the combination of oil with metal oxide for the preparation of bionanocomposite is not easy and straightforward. Hence, the requirement of a polymeric medium acts a precursor to support the preparation of interested bionanocomposite is always needed [32].

Polyurethane (PU), is a moldable biocompatible strong polymer that has a vast range of applications. Furthermore, it serves as a strong stabilizer and prevents the aggregation of nanoparticles as a result of repulsive forces originating from the hydrophobic carbon chains that are spread into the organic solvents and intermolecular interaction [33]. Likewise, it plays important role in the stabilization of synthesized nanomaterials. It was chosen as an efficient binding and protective agent on the basis of its remarkable chemical and physical characteristics including; adhesive, chemically inert, optical transparency, and potential stability [34]. Moreover, PU has displayed excellent properties in many areas such as biomaterials, biosensors, and biomedical [35]. The biocompatibility of PU makes it a good choice in biomedical engineering applications. Additionally, the Food and Drug Administration (FDA) has approved PU as the safest and stable polymer for pharmaceutical and biological studies.

Considering the aforementioned individual distinctive features of nutmeg oil, ZnONPs, and PU, this study describes the synthesis of polymeric nutmeg oil/PU decorated with ZnONPs for the first time. The literature survey addressed a few reports about nutmeg oil-based nanoparticles blend systems. Pranati et al. (2003), addressed the use of nutmeg oleoresin for the preparation of silver nanoparticles and its antimicrobial potential towards oral pathogens [36]. Another study conducted by Mousavi et al. (2019) reported the synthesis of well-decorated super-magnetic Fe_3_O_4_-MgO nanoparticles using nutmeg essential oil with promising antimicrobial properties [37]. The results of the previous reports suggested that nutmeg oil has significant potential to be incorporated into the polymeric matrix to form bionanocomposite with advanced biological properties. Thus, the objective of the present study is the preparation of nutmeg oil/PU/ZnO bionanocomposite using nutmeg oil, PU, and ZnONPs and evaluate its immunomodulatory, antimicrobial, and antioxidant potential.

## 2. Materials and Methods

### 2.1. Botanical Material

Commercial samples of dry nutmeg seeds (1.0 kg) were acquired from a supermarket of Riyadh, Saudi Arabia, during September 2020, and identified by the taxonomist professor Dr. Mohamed Yousef at the Pharmacognosy Department of the King Saud University. Seeds of nutmeg were ground into a fine powder using a domestic blender and sieved into various particle size (0.34, 0.65, and 1.54 mm) grades.

### 2.2. Extraction of Nutmeg Oil

Nutmeg oil was extracted by high vacuum distillation according to the previously reported procedure [38]. The powdered seeds of nutmeg (900 g) were mixed with distilled water twice its volume (2.0 L) to get a concentrated slurry which was frozen in liquid nitrogen prior to use. The sample was then subjected to 8 h vacuum distillation. The obtained distillate was fractionated with diethyl ether (3 × 2.0 L) to get the volatile components. The fraction was freed from the solvent by a slow stream of nitrogen to yield the concentrated oil. The essential oil collected was dried over anhydrous sodium sulfate and immediately weighed after the collection. The obtained essential oil was stored in dark-colored sealed glass tubes at 4 ºC and the percentage yield of essential oil was calculated. 

### 2.3. GC/MS Analysis of Essential Oil

The nutmeg seeds oil analysis was carried out by a GC/MS (6890-Packard Hewlett series) equipped with a selective mass detector (J&W Scientific, Agilent Tech., CA, USA) and provided with an HP-5 MS capillary column (5% phenyl methylsiloxane, 30 m × 0.25 µm × 250 µm). The operating conditions were: temperature of the column was organized from 50 to 250 °C at a rate of 5 °C/min, held as initial temperature followed by 250 °C for 5 min and finally to 280 °C at a rate of 10 °C/min, held for 20 min for final temperature; Injector and interface temperatures were programmed at 210 °C and 230 °C, respectively; the applied carrier gas was helium at 1.0 mL/min flow rate and 0.5 µL sample volume was injected. The guarantee of reproducibility of experiment was acquired by performing sample analysis in triplicates. The peaks were tentatively identified by comparing the fragmentation pattern of mass and retention time (R_t_) with those of reference compounds available, from the standard spectral library (Wiley/NIST 2008) available in the instrument and literature data.

### 2.4. Preparation of Myristica Fragrans (Nutmeg) Biomass

Nutmeg seeds were collected and washed with distilled water to remove any contaminants, and dried for one week. The dried seeds were finely powdered and sieved to obtain a fine, homogeneous powder. Approximately, 10 g of powdered nutmeg seeds were soaked in 100 mL of distilled water and subjected to continuous magnetic stirring for 1 h at 95 °C. The extract was filtered using Whatman paper No. 41 (pore size 25 μm) and centrifuged at 3500 rpm for 20 min to obtain the clear aqueous extract. The final extract was stored in a glass bottle in the refrigerator at 4 °C until further use. 

### 2.5. Synthesis of ZnO Nanoparticles Using Nutmeg Biomass

The nutmeg seeds aqueous biomass was used to synthesize the ZnONPs. Briefly, 50 mL of 1.0 × 10^−3^ mol L^−1^ of zinc acetate solution was mixed with 25 mL of nutmeg aqueous extract at 60 °C for 1 h under continuous magnetic stirring. About 5.0 mL of 0.1 mol L^−1^ of sodium hydroxide was added dropwise to the reaction mixture to precipitate out the ZnONPs. The precipitate was centrifuged at 5000 rpm for 20 min, filtered through Whatman paper No. 41 (pore size 25 μm), and air oven-dried at 80 °C for 3 h. The obtained ZnONPs were calcined at 500 °C for 4 h and stored in a tight glass bottle prior to use.

### 2.6. Synthesis of Polymeric Nutmeg Oil/PU/ZnO Bionanocomposite film

A polymeric nutmeg oil/PU/ZnO bionanocomposite film was prepared by mixing dropwise 10 % (*w*/*v*) of nutmeg oil to the PU solution/ZnONPs blend. Briefly, 20 g of PU dissolved in 100 mL deionized water under continuous magnetic stirring for 24 h to obtain a homogenous polymeric solution. Afterward, 10 % of ZnONPs was added to the polymeric solution and vigorously mixed under continuous magnetic stirring for 12 h at room temperature. A mixture of nutmeg oil and Tween 80 (0.20 g/g of nutmeg oil) was added to the film-forming solution at different concentrations (5%, 10%, and 15% *w*/*v* on the basis of neat film solution). The change in color from pale yellow to dark brown confirmed the formation of nutmeg oil/PU/ZnO bionanocomposite solution. The obtained solution was homogenized at 2500 rpm for 10 min using (Ultra-Turrax homogenizer, Deutschland, Germany) and the resulting solution was degassed for 30 min under vacuum to free from all bubbles. Finally, the films were developed by casting process by spreading 60 mL of dispersion solution over a Teflon plate and dried at 25 ± 2 °C for 24 h. The dried films were removed from the teflon plate and preconditioned in as desiccator containing a saturated magnesium nitrate solution at ambient temperature until further use. 

### 2.7. Characterization of ZnO Nanoparticles and Bionanocomposite

The formation of ZnONPs and nutmeg oil/PU/ZnO bionanocomposite was confirmed by various spectroscopic methods. UV–vis spectroscopy (Ultrospec 2100^®^ pro, Biochrom, UK), Fourier-transform infrared (FTIR, 4000–400 cm^−1^ range) spectroscopy, and X-ray diffraction (XRD, D/MAX 2500-X-ray diffractometer, Rigaku Corporation, Tokyo, Japan) were performed to study the crystalline structure of the formed nanoparticles. XRD analysis was conducted to confirm the presence of ZnONPs in the bionanocomposite using Bragg angles ranging from 15° to 75°, voltage 30 kV, and 45 mA. Scanning electron microscope and transmission electron microscope (SEM, JSM-7610Fand TEM, JEM-1400 Plus, JEOL, Pleasanton, CA, USA) were used to examine the morphology of the surface and particle size of the pre-synthesized nanomaterials.

### 2.8. Thermal Stability and Hydrolytic Degradation of Bionanocomposite Film

The thermal stability of the fabricated nutmeg oil/PU/ZnO bionanocomposite film was conducted by thermogravimetric analysis, using thermogravimetric analyzer (TGA, Seiko Exstar 6300, Tokyo, Japan). The experiment was conducted by heating 5 mg of targeted bionanocomposite from room temperature to 600 °C under constant flow of nitrogen gas (50 cm^3^/min) at 25 °C/min heating rate to get thermogravimetric analysis (TGA) without oxidative decomposition [39]. The TGA value differentials were applied to obtain the TGA derivative (TGAD), which was calculated by forward finite difference procedure:

$$\mathrm{TGAD}=\left(\frac{w_{t+\Delta t}-w_{t}}{\Delta t}\right)$$

where *w_t_*_+∆*t*_ and *w_t_* express the weight residual of targeted sample at *t* + ∆*t* time and *t*, respectively. The ∆*t* denotes the reading of time interval for residual investigated sample weight. The bionanocomposite was also tested for hydrolytic degradation by adjusting the temperature and pH of the system according to per ASTM F1635-11 method [40]. The hydrolytic degradation estimation of bionanocomposite was conducted for 8 weeks in 10 mL of phosphate-buffered saline (PBS, pH 7.4) solution and heated at 37 °C in the water bath. The removal of the film from the control medium was performed at 2nd, 4th, 6th, and 8th week time intervals, and dried at 60 °C in the oven. Consequently, the weight reduction in targeted bionanocomposite film was measured via gravimetric analysis along with a degradation test. Then, before initiating the experiment time zero was adjusted and the measurements were recorded at each withdrawal time of the film. At the 8th week, no weight loss was observed due to extreme degradation of targeted bionanocomposite film. The weight loss of bionanocomposite film was calculated in triplicates using the following equation:

M = M_0_ − M_f_

where M (weight loss), M_0_ (mass of the film) and M_f_ (mass of the film at different time intervals).

### 2.9. Immunomodulatory Activity

#### 2.9.1. Cell Culture

Macrophage RAW 264.7 cells were grown in RPMI-1640 medium supplemented with 10% of fetal bovine serum and 1% of antibiotics (penicillin-streptomycin) with 5% CO_2_ supply at 37 °C in an anaerobic incubator. The RAW 264.7 cell concentration was diluted to 5 × 10^4^ cells/well density in the 96-microwell plate to evaluate the effect of samples on the cell proliferation and the pinocytosis potential. The level of cytokines was measured in a 24-well plate with cell density 1 × 10^5^ cells/well placed in an anaerobic incubator with 5% CO_2_ flow at 37 °C. After 12 h incubation, addition of treatment, control, and various concentrations of samples was carried out onto the well plate and incubated for another 24 h at 37 °C. Blank and negative control DMSO (100 µL), LPS control group (2 µg mL^−1^), and different concentrations of investigated samples (30, 60, 120, 240, 480, and 960 µg mL^−1^) were included in treatments [41].

#### 2.9.2. Cell Proliferation and Pinocytosis Effects Assay

The effect of samples on the cell proliferation and pinocytosis potential of RAW 264.7 cells were performed by obeying the CCK-8 procedure using cell counting kit-8 (AbMole) and the neutral red staining procedure, respectively [42]. The experiment was performed six times for each sample using a 96-well plate.

#### 2.9.3. Estimation of IL-6, TNF-α, IL-10 Cytokines and Nitric Oxide

ELISA kits (Multi-Science Biotech, Zhejiang, China) were used to determine the levels of different cytokines such as IL-6, TNF-α, and IL-10 in the cell culture supernatant. The Griess method was applied to measure the concentration of nitric oxide (NO) [43]. Sodium nitrite standard solution was applied for the calibration graph. Each experiment was repeated in triplicates using a 24-well plate.

#### 2.9.4. RT-qPCR Determination of Cytokines Levels of mRNA

RNeasy Mini Kit (Qiagen, GER) was used to extract the total RNA from RAW 264.7 macrophages. The RNA extracted was reverse transcribed by applying the standard cDNA PrimeSript 1st synthesis kit (Thermo k1622, Carlsbad, CA, USA). The cytokines (IL-6, TNF-α, and IL-10) genes expressions were measured by applying the SYBR Green Master FastStart Universal (ROX) (Roche, CH) equipped with Real-Time PCR Agilent-Mx3000p system (Agilent, Santa Clara, CA, USA). The quantitative analysis of other genes was performed by applying β-actin genes as references. RT-PCR studies were conducted in triplicates.

### 2.10. Antioxidant Activity

DPPH (1,1-diphenyl-2-picrylhydrazyl) and ABTS (2,20-azino-bis[3-ethyl benzo thiazoline-6-sulphonic acid]) free radical scavenging assays were performed to assess the antioxidant potential of nutmeg oil, ZnONPs, and bionanocomposite.

#### 2.10.1. DPPH Assay

The DPPH free radical scavenging effect of nutmeg oil, ZnONPs, and bionanocomposite were determined by obeying previously described Khan et al. standard methods [44]. In brief, DPPH (0.1 mM), three different concentrations of investigated samples (25, 50, 100 µg mL^−1^), and ascorbic acid were dissolved in methanol before the experiment. Then, 50 µL of DPPH solution was treated separately with different concentrations of each test sample and ascorbic acid solution in a 96-well microplate. The reaction mixture was mixed well and placed in the dark for 30 min. After incubation, absorbance at 520 nm was noted against the blank (methanol) on a BioTek microplate reader (Biotech instruments, Incorporated, Highland Park, CA, USA). The scavenging ability was determined using the following equation:

$$\%\;\mathrm{DPPH}\;\mathrm{free}\;\mathrm{radical}\;\mathrm{potential}=\frac{Cr-Tr}{Cr}\;\times 100$$

where *Cr* and *Tr* represent the absorbance of control and test sample, respectively.

#### 2.10.2. ABTS Assay

The ability of test samples to scavenge ABTS free radical was evaluated by following Qiu et al. standard method with slight modification [45]. Briefly, two individual stock solutions of ABTS (7.4 mM) and potassium persulfate (2.6 mM) were prepared. Afterward, equal amounts of stock solution (ABTS and potassium persulfate) were mixed to prepare the working ABTS solution and placed undisturbed for 12 h under dark conditions. In a 96-microwell plate, 125 µL of ABTS working solution was reacted with 10 µL of different concentrations (25, 50, 100 µg mL^−1^) of investigated samples and reference standard (ascorbic acid). The reaction mixture was incubated at ambient temperature for 2 h in the dark and observed for the color change. Finally, at 520 nm the absorbance of the reaction mixture was recorded in contrast to the blank (methanol) using a microplate reader. The scavenging % potential was calculated by applying the above-mentioned equation.

### 2.11. Statistical Analysis

All the experiments were conducted in triplicates. The resultant data were represented as mean ± SD and tested for their statistical significance of difference with ANOVA. The *p* < 0.05 was considered significant statistically by applying IBM SPSS Modeler 18.0. software (Agilent, Santa Clara, CA, USA). The standard error deviation for each sample was represented by error bars on graphs.

## 3. Results and Discussion

### 3.1. Chemical Analysis of Isolated Essential Oil

The hydro-distillation of grounded nutmeg seeds yielded golden-brown oil. The chemical composition of isolated nutmeg oil analyzed by GC-MS is presented in Table 1.

**Figure 1:**
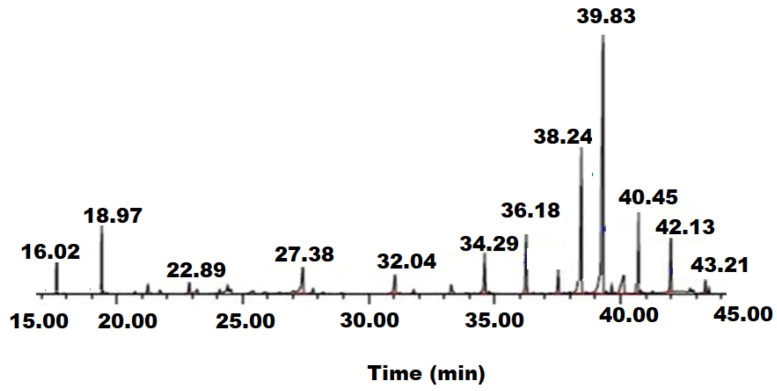
GC-MS chromatogram of nutmeg oil chemical components.

In total, 31 components were identified in nutmeg oil. The oil is comprised of terpene hydrocarbons (such as pinene, camphene, myrcene, phellandrene, carene, limonene, and camphene), terpene derivatives (including α-cubebene, linalyl butanoate, terpinen-4-ol, copaene, anthrone, asarone, and safrol), and myristicin as the main constituents. The composition of myricitin, terpene hydrocarbons, and derivatives of terpenes were 33.25%, 40.58%, and 8.45%, respectively, revealing that myricitin is the major component of the oil (Figure 1).

### 3.2. Characterization of ZnONPs

**Figure 2:**
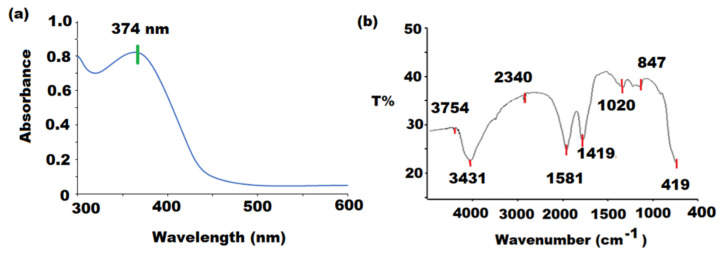
(**a**) UV-Vis and (**b**) FT-IR spectra of ZnONPs at 374 nm and 4000 to 400 cm^−1^.

The ZnONPs were synthesized by mixing zinc acetate solution with aqueous nutmeg extract at 60 °C for 1 h under contentious magnetic stirring. The light brown precipitation confirms the formation of ZnONPs. The characterization of ZnONPs was conducted by spectroscopic and microscopic investigations. The UV–Vis spectrum of ZnONPs was measured at 200–400 nm wavelength range. A broad peak at 374 nm was observed in the UV–Vis spectrum of ZnONPs (Figure 2a). The increased shift of UV-peak could be attributed to nanoparticles aggregation due to ZnO nano-assemblage and the presence of different secondary metabolites that interaction with zinc acetate in the solution might be the reason for the reduction in zinc acetate precursor to ZnONPs. The observed results were consistent with the previously reported studies [46]. The reduction and stabilization in the stretching and bending vibrational frequencies of the components that are persuading reduction and stabilization in ZnONPs were measured within the 4000–400 cm^−1^ range by FT-IR spectroscopy (Figure 2b). The vibration peak due to asymmetric stretching at 3754 cm^−1^ was observed in Zn(OH)_2_ crystal structure. Two stretching vibration peaks at 3431 cm^−1^ and 2340 cm^−1^ for the hydroxyl group of water were noticed [47]. The peaks that appeared at 2370 cm^−1^, 1581 cm^−1^, 1419 cm^−1^, and 1020 cm^−1^ were assigned to carbonyl, hydroxyl, and O-H bonds. Two significant absorption peaks at 847 cm^−1^ and 419 cm^−1^ confirmed the formation of Zn-O vibration stretching bond in the zinc hydroxide molecule.

**Figure 3:**
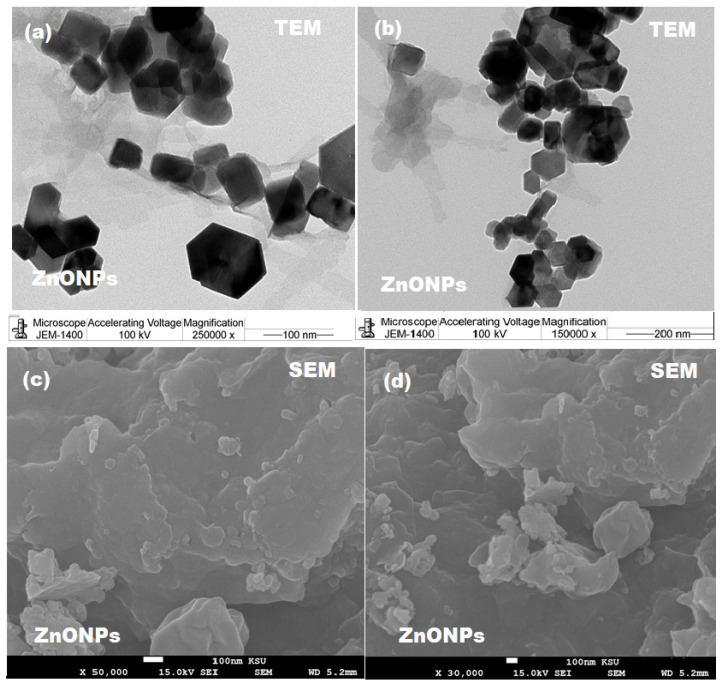
(**a**,**b**) TEM images at ×250,000 and ×150,000 magnification and (**c**,**d**) SEM images at ×50,000 and ×30,000 magnification of ZnONPs.

The formation of ZnONPs was further confirmed by TEM and SEM microscopy. The homogeneous crystal structures without pores of pre-synthesized ZnONPs were confirmed at different magnifications (×250,000 and ×150,000) under TEM microscope and the particles were found in the range of 10–100 nm (Figure 3a,b). At two different magnifications (×50,000 and ×30,000) SEM images were monitored and a hexagonal lamellar smooth structure with 100 ± 5 nm length and 25 nm thickness was observed (Figure 3c,d).

**Figure 4:**
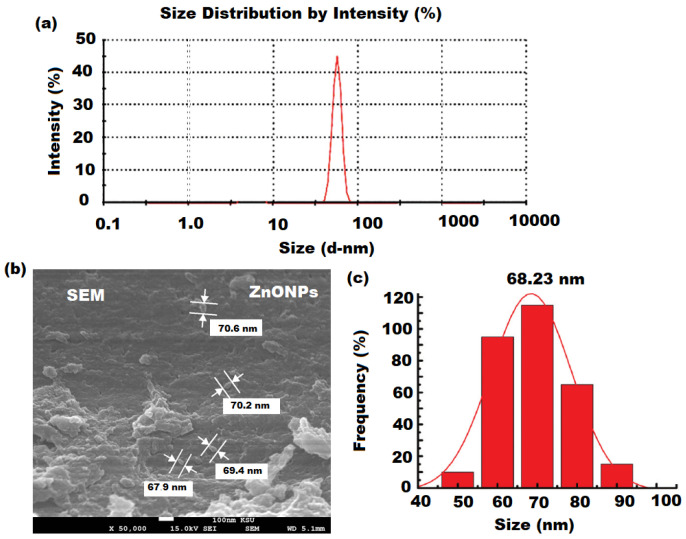
(**a**) DLS analysis (**b**) SEM, and (**c**) particle size distribution of ZnONPs.

The average diameter of nutmeg mediated ZnONPs was measured by Dynamic Light Scattering (DLS) and was found to be 68.23 nm. The results showed that the synthesized nanoparticles were ultrafine and less than 100 nm in diameter. The reduction in particle size might be due to the existence of various phytoconstituents in the aqueous extract of nutmeg. Additionally, the DLS analysis displayed that the formed nanoparticles possess fairly well-defined dimension (Figure 4a–c).

**Figure 5:**
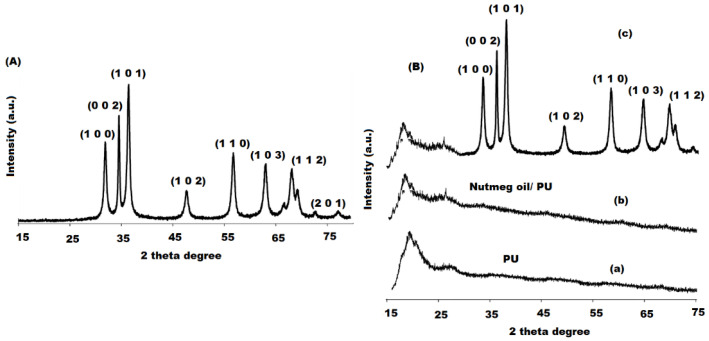
XRD spectrum of (**A**) ZnONPs and (**B**) plain PU (**a**), nutmeg oil (**b**) and bionanocomposite (**c**) in 20–80 wave number range.

The crystal and phase structure of the bio-synthesized of ZnONPs were studied by XRD analysis. A specific line broadening of the XRD peaks indicates that particles in the nanoscale were involved in the prepared material. The XRD patterns showed various diffraction peaks at 31.84° (1 0 0), 34.52° (0 0 2), 36.38° (1 0 1), 47.64° (1 0 2), 56.70° (1 1 0), 63.07° (1 0 3), and 68.10° (1 1 2) were assigned as hexagonal wurtzite phase of ZnO with *a* = *b* = 0.324 nm and *c* = 0.521 nm lattice constants (JPCDS card number: 36-1451) [48]. Moreover, it was confirmed that the formed ZnONPs were impurity-free as no additional peaks other than ZnO peaks were recorded in the XRD pattern (Figure 5A). The presence of regular succession in the atomic planes suggests that the nanocrystallites are uniform structurally and crystalline without amorphous phase. The diameter of pre-synthesized nanoparticles was established by Debye–Scherrer formula: [49].

*d* = 0.89*λβ*cos*θ*

where 0.89, *λ*, *θ*, and *β* were Scherrer’s constant, the X-rays wavelength, Bragg diffraction angle, and the full width at half-maximum (FWHM) of the diffraction peak with respect to the plane (1 0 1). FWHM was applied to determine the crystalline size of a more intense peak and the calculated value of crystallite size D = 74.52 ± 2.3 nm with respect to (1 0 1) plane at 36.38° by applying Scherrer’s formula [50].

The nutmeg oil/PU/ZnONPs bionanocomposite film was fabricated by treating nutmeg oil with ZnONPs in the presence of PU matrix at room temperature. The formation of bionanocomposite was confirmed by the change in color from pale yellow to dark brown. The bionanocomposite characterization was performed by applying various techniques such as XRD and SEM. XRD pattern confirms the dispersion of nutmeg oil and ZnONPs in the polymeric PU medium with Cu kα radiation (*λ* = 1.540 Å), 60 mA voltage, and 30 kV over 15°–75° Bragg angles range. The XRD spectrum of plain PU showed a broad peak, suggesting its amorphous nature (Figure 5(Ba)) [51], whereas nutmeg oil exhibited a broad peak with high intensity near ~30° (Figure 5(Bb)). However, the XRD spectrum of nutmeg oil/PU/ZnONPs bionanocomposite expressed many distinct peaks for ZnO (1 0 0), (0 0 2), (1 0 1), (1 0 2), (1 1 0), (1 0 3), and (1 1 2) at hexagonal wurtzite phase of ZnO at 31.84°, 34.52°, 36.38°, 47.64°, 56.70°, 63.07°, and 68.10°, respectively (Figure 5(Bc)), which confirms the good distribution of ZnONPs on the surface of bionanocomposite film.

**Figure 6:**
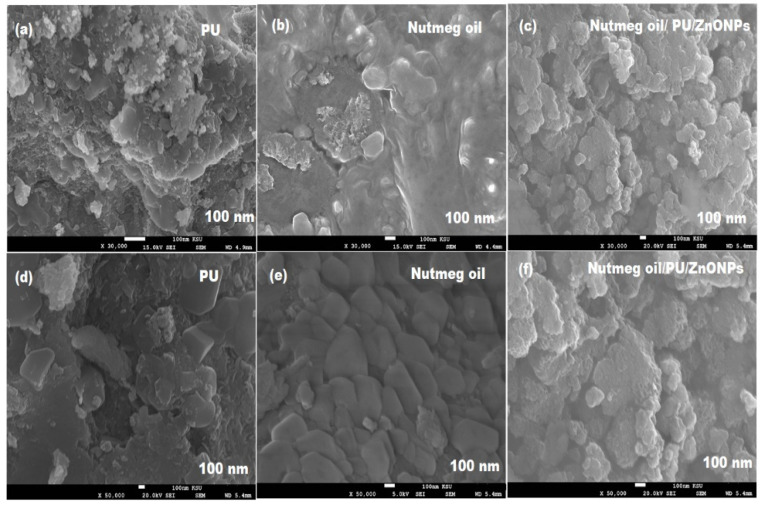
SEM images of (**a**,**d**) plain PU, (**b**,**e**) nutmeg oil, and (**c**,**f**) nutmeg oil/PU/ZnNPs bionanocomposite at ×30,000 and ×50,000 magnification.

The morphological shape of plain nutmeg oil and pre-fabricated bionanocomposite was further confirmed under SEM at different magnifications (×30,000 and ×50,000). Figure 6a–f shows the surface morphology of PU, nutmeg oil, and nutmeg oil/PU/ZnONPs. The SEM images of PU at ×30,000 and ×50,000 magnifications display a smooth surface with a small diameter (Figure 6a,d). While the plain nutmeg oil shows a distorted shape with irregular edges and tiny pores on the coagulant surface for the oil droplets under the same magnification (Figure 6b,e). However, the SEM images of the synthesized bionanocomposite film show the good distribution of ZnONPs in the nutmeg oil and PU medium with 80 ± 5 nm particle size at a similar magnitude (Figure 6c,f). The particle size distribution of PU solution containing 10% (*w*/*v*) nutmeg oil was also examined. The diameter particle size distribution was found in the range of 50 ± 10 nm.

**Figure 7:**
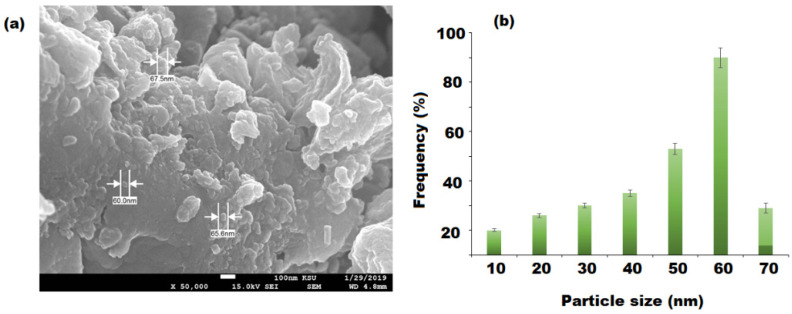
SEM image of (**a**) bionanocomposite containing 20 w% of nutmeg oil and (**b**) the particle size distribution and diameter of ZnONPs.

Furthermore, bionanocomposite showed a homogeneous distribution with a 60 nm particle size diameter range (Figure 7a,b). The appearance of ZnONPs might be responsible for the soft surface of the fabricated nutmeg/PU/ZnONPs bionanocomposite and indicated the successful use of nutmeg oil and PU for the biogenic synthesis targeted bionanocomposite.

**Figure 8:**
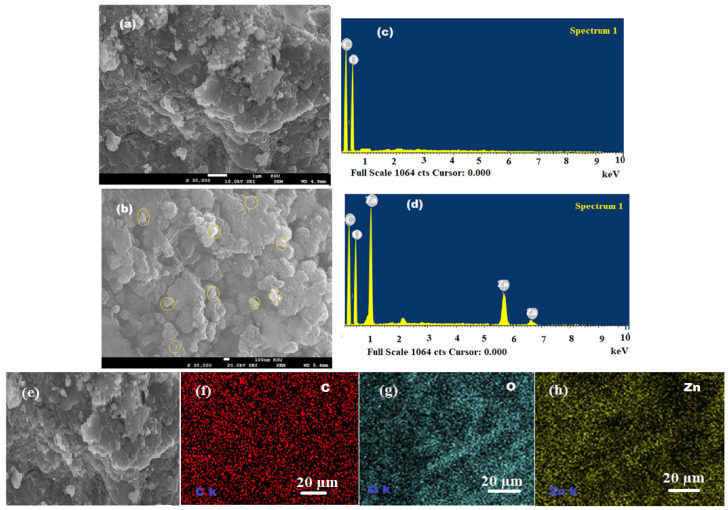
(**a**,**b**) SEM images of PU and ZnONPs dispersed in the nutmeg oil/PU/ZnONPs polymeric bionanocomposite, (**c**,**d**) EDX spectra of plain PU and bionanocomposite, and (**e**–**h**) elemental distribution C-red, O-blue, and Zn-yellow in the EDX mapping of bionanocomposite.

SEM equipped with EDX was used to investigate the morphological shape of the virgin PU and bio-synthesized bionanocomposite by EDX analysis. The presence of ZnONPs on the surface of bionanocomposite suggested their good distribution in the polymeric medium (Figure 8a). Two significant signals have appeared for C and O of PU (Figure 8b). However, the pre-synthesized bionanocomposite exhibited the occurrence of three significant signals for C, O, and Zn (Figure 8c,d). There was no other elemental impurity noticed in the bionanocomposite structure. Hence, EDX results confirmed the successful fabrication of bionanocomposite and there was excellent distribution of ZnONPs and nutmeg oil in the polymeric PU matrix. Moreover, the EDX mapping images revealed that the components of bionanocomposite film are homogeneously distributed with some agglomeration of ZnONPs with the increase in ZnO content (Figure 8e–h) [39].

### 3.3. Bionanocomposite Thermal Stability and Hydrolytic Degradation Studies

**Figure 9:**
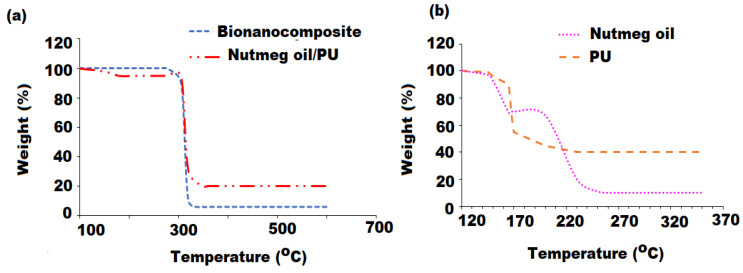
Thermogravimetric analysis of: (**a**) nutmeg oil/PU/ZnONPs bionanocomposite and nutmeg oil/PU and (**b**) nutmeg oil and PU.

The thermal stability of nutmeg oil/PU, nutmeg oil/PU/ZnO bionanocomposite, and PU film was investigated by comparing graphs of TGA and their TGA and TGAD with those of nutmeg oil and PU films as depicted in Figure 9a,b. The weight loss pattern in the TGA and the TGAD graphs were used to evaluate the thermal decomposition of the films that clearly showed the maximum decomposition temperature (*T*_max_) at each step of thermal degradation. The nutmeg oil/PU/ZnO bionanocomposite film displayed multistep thermal degradation due to the behavior of thermal decomposition of phytoconstituents present in the nutmeg oil and ZnONPs of bio-synthesized film. As illustrated in the TGA graphs, the thermal decomposition process of PU and nutmeg oil occurred in three steps. The initial degradation of bionanocomposite film started with loss of 10–15% moisture content at around 100 °C from the surface of the film [52]. The second thermal degradation begins at 168 and 120 °C and showed maximum degradation rate at 230 and 165 °C for the nutmeg oil and polymeric PU films, respectively, which could be due to the volatilization of constituents of the nutmeg oil. The major decomposition occurred at 305 and 320 °C with maximum decomposition rate at 350 and 370 °C is mainly attributed to the degradation of long biopolymeric chain. Assumably, the ZnONPs has a twin effect on the bionanocomposite, i.e., catalytic effect towards the nutmeg oil and polymeric medium that lower the thermal stability and also serves as a barrier to increase the thermal stability. The obtained results indicated that the thermal stability of pre-synthesized bionanocomposite film was up to 370 °C and higher in contrast to the individual components (nutmeg oil, PU, and ZnONPs) of bionanocomposite film due to inherent characteristics of samples used and strong intermolecular interaction [53]. However, the weight loss in the hydrolytic degradation of bionanocomposite showed different pattern. The obtained results showed no weight loss of bionanocomposite at the start of the experiment. Whereas, in the 4th and 6th week of experiment there was 6.78% and 14.34% of weight loss in bionanocomposite film, respectively (Table 2). It may be hypothesized that the addition of ZnONPs and PU affected the degradation by promoting the penetration of water into the system that has led to the polymeric chain diminution and increased the disintegration of the medium.

### 3.4. RAW 264.7 Cell Proliferation Measurement

**Figure 10:**
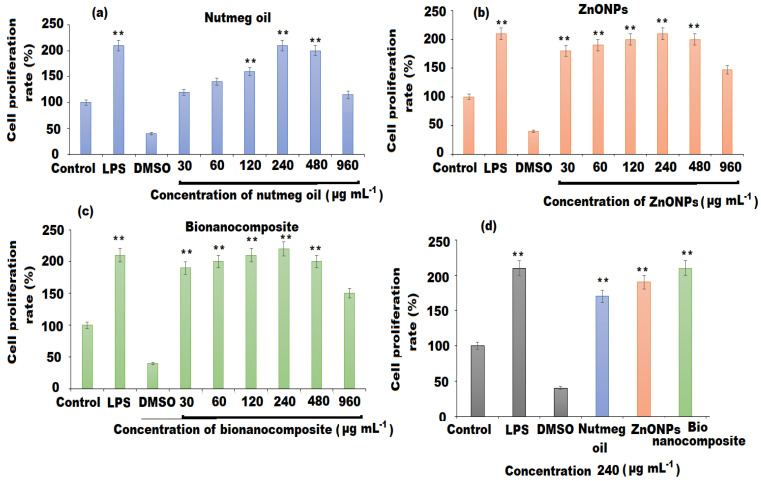
Cell proliferation rate of: (**a**) nutmeg oil (**b**) ZnNPs, (**c**) bionanocomposite, and (**d**) comparative analysis. The mean values and standard deviation (SD) were represented by bars and error bars, respectively for three independent determinations (n = 3). (**) indicate (*p* < 0.01) compared to the control.

Immunoregulation activities of nutmeg oil, ZnONPs, and bionanocomposite were evaluated in the RAW 264.7 cell model. Different concentrations of test samples (30, 60, 120, 240, 480, and 960 µg mL^−1^) were chosen. LPS (2 µg mL^−1^) as a positive control that could activate the proliferation rate (*p* < 0.01), while DMSO (100 µL) was used as a negative control that does not affect cell proliferation. The results revealed that nutmeg oil, ZnONPs, and bionanocomposite could increase the cell proliferation rate of RAW 264.7 (*p* < 0.01) in a concentration-dependent manner, whereas few cytotoxic effects were noticed at higher concentrations (Figure 10). At 240 µg mL^−1^ concentration, all the test samples demonstrated high proliferation rates, with pronounced effect displayed by bionanocomposite followed by ZnONPs, and moderate effects were observed for nutmeg oil (Figure 10a–d). The strong potential of bionanocomposite could be attributed to: (1) the synergistic effects of nutmeg oil, PU, and ZnONPs; and (2) the interaction of the chemical components of the essential oil with ZnONPs co-existing in the bionanocomposite, contributing to the high proliferation rates of RAW 264.7 cells.

### 3.5. Phagocytic Effect and Nitric Oxide Release of Macrophages

**Figure 11:**
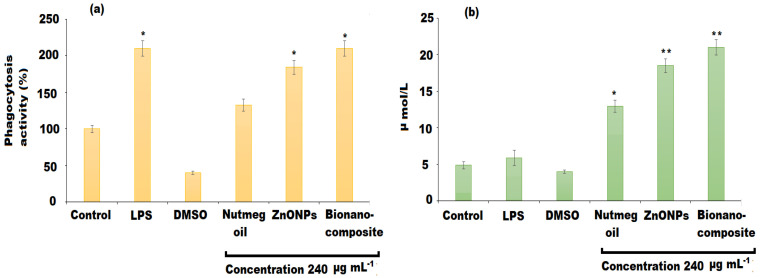
Effects of nutmeg oil, ZnONPs, and bionanocomposite on (**a**) phagocytosis activity, and (**b**) production of NO. The mean values and standard deviation (SD) were represented by bars and error bars, respectively for three independent determinations (n = 3). (*) and (**) indicate (*p* < 0.05) and (*p* < 0.01) compared to the control.

Phagocytosis is the most remarkable feature for the activation of macrophages and is a crucial barrier for the host to amplify the innate immune system. Nitric oxide contributes a key role in the anti-inflammatory response and inhibition of the proliferation of cancer cells [54]. The activation of macrophages causes increase secretion of nitric oxide. The effects of nutmeg oil, ZnONPs, and bionanocomposite on the phagocytosis activity and secretion of nitric oxide in RAW 264.7 macrophages were shown in Figure 11. The results showed that only LPS and bionanocomposite elevated the phagocytic ability potentially (*p* < 0.05), whereas ZnONPs could significantly promote the production of nitric oxide (*p* < 0.01) (Figure 11a,b). However, nutmeg oil only exhibited a moderate phagocytic effect.

### 3.6. Estimation of Cytokines and mRNA Expression Levels

**Figure 12:**
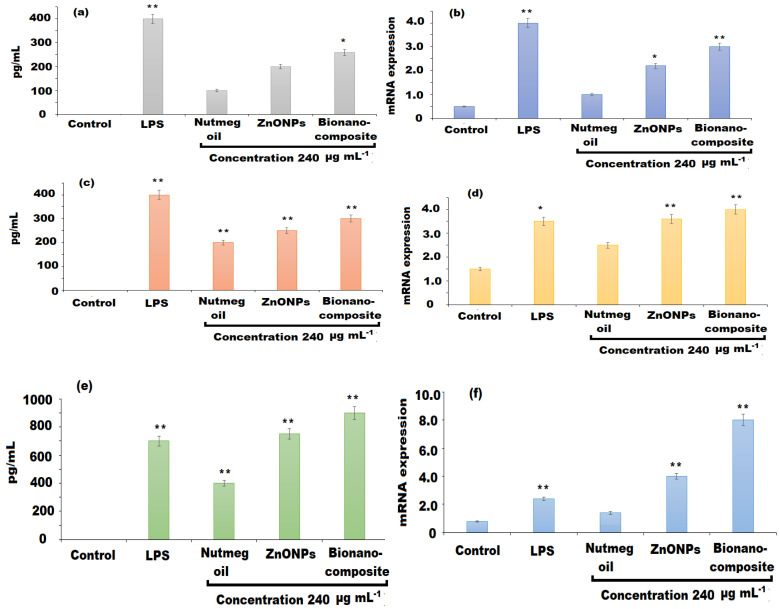
Effects of nutmeg oil, ZnONPs, and bionanocomposite on the levels of cytokines and mRNA expression (**a**,**b**) IL-6, (**c**,**d**) TNF-α, and (**e**,**f**) IL-10. (*) and (**) indicate (*p* < 0.05) and (*p* < 0.01) compared to the control.

The immune cells after the intrusion discharge of various cytokines including TNF-α, IL-6, and IL-10, which ameliorate their capability to defend cancer cells and pathogenic infections [55]. The cytokine IL-10 has also shown remarkable anti-inflammatory effects. In the current study, the effects of nutmeg oil, ZnONPs, and bionanocomposite on the cytokines TNF-α, IL-6, and IL-10 was assessed by ELISA analysis (Figure 12a–c). The results revealed that bionanocomposite significantly promoted the IL-6 (*p* < 0.05) production, followed by ZnONPs as compared to the control group, whereas nutmeg oil showed no significant difference for the production cytokines except LPS (*p* < 0.01). As shown in Figure 12c, nutmeg oil showed no influence on the secretion of TNF-α, whereas bionanocomposite and ZnONPs significantly activated the production of TNF-α (LPS, bionanocomposite, and ZnONPs to 60, *p* < 0.05, and *p* < 0.01, respectively). Moreover, all the investigated samples exerted a significant increase in IL-10 cytokine level (*p* < 0.01) as compared to the control group at 240 µg mL^−1^ concentration. In addition to cytokines production, mRNA expression levels of IL-6, TNF-α, and IL-10 were also evaluated by RT-qPCR analysis (Figure 11a–c). The results showed that the bionanocomposite potentially improved the mRNA expression of IL-6 and TNF-α (*p* < 0.01), while the ZnONPs expressed an increase in mRNA expression of IL-10 (*p* < 0.01) only. However, nutmeg oil showed an increase in mRNA expressions of cytokines in traces. The potential effect of bionanocomposite film on the production of cytokines may be attributed to synergistic effects of nutmeg oil, ZnONPs, and PU in the bionanocomposite, and could facilitate the better mRNA expressions of IL-6 and TNF-α (Figure 12b,d). Moreover, the interaction between chemical components of the oil and ZnONPs could affect their affinity surface receptors of the immune cell, resulting in different immune effects.

### 3.7. Correlation of Immunomodulatory Activity and Chemical Structure

The terpenes and polyphenolics from several medicinal plants modulate the immune effects through various ways, including macrophage function by initiating the release of immune cytokines (IL-6, TNF-α, IL-10, and IL-β), and cellular, hormonal, and mucosal immunity. The literature survey has addressed that the different structures, functionalities, and vast range of molecular weight distribution might be the contributing characteristics for their immunomodulatory effect [56]. Abaci et al. reported that carboxyl group of triterpenes, isolated from *Cephalaria sumbuliana,* was involved in the activation of HEK293 cells [57]. While according to an in vitro study, Askar et al. showed that ZnONPs play a crucial role in the immunomodulatory activity by enhancing the parent activity of plant extract [58]. However, recent progress in tissue engineering scaffolds based on polyurethane and polyurethane hybrids have reported that polyurethane and modified polyurethane showed promising immune activities by the incorporation of active moieties to the structure [59]. Moreover, the long carbon chain in PU also plays a critical role in the enhancement of immunomodulatory potential. In the current study, bionanocomposite possess relatively high immunomodulatory potential, which might be attributed to the combined effects of phytoconstituents of nutmeg oil, ZnONPs, and PU. However, nutmeg and ZnONPs contain secondary metabolites in their structure. Hence, the bionanocomposite with the structural modification is the best candidate for immunomodulatory activity in the future.

### 3.8. Antioxidant Activity

The antioxidant activity of plain oil, ZnONPs, and bionanocomposite at different concentrations (25, 50, 100 µg mL^−1^) were tested on *DPPH* free radical [60] and the results were summarized in Table 3. All the tested samples were free radical scavengers. However, the strongest scavenging potential was shown by bionanocomposite followed by ZnONPs compared to nutmeg oil. The DPPH free radical scavenging activity of investigated samples increases with the increase in dose concentration. At 25–100 μg mL^−1^ concentrations, the bionanocomposite and ZnONPs showed 52% to 68% with an average IC_50_ value 0.28 ± 0.22 and 0.55 ± 0.14 scavenging activities, respectively. However, nutmeg oil showed 40% with IC_50_ value 0.57 ± 0.32 scavenging activity. The antioxidant potential of the three tested samples was found to be lower than the reference standard ascorbic acid (82%) at the same concentration.

In the ABTS assay, the capability of bionanocomposite to reduce ABTS to free radical ABTS^+^ was significantly higher than ZnONPs as well as nutmeg oil at 25–100 μg mL^−1^ concentrations by 62% to 72% (IC_50_ value 0.49 ± 0.36). Some previous studies stated that oil-based bionanocomposite and ZnONPs possess potential antioxidant activity [61]. The nutmeg oil used in the composition of bionanocomposite is an excellent source of polyphenolics that are the main components responsible for the antioxidant properties [62]. Moreover, the presence of polymeric matrix and ZnONPs has significantly enhanced the antioxidant properties of bionanocomposite due to the compatible biological and chemical features of ZnONPs, PU, and the presence of natural phenolics in the nutmeg oil.

## 4. Conclusions

The above research study described the eco-friendly, cost effective, and non-toxic amiable fabrication of nutmeg oil/PU/ZnO bionanocomposite film. The synthesis involves the utilization of nutmeg oil, PU, and ZnONPs prepared from the aqueous extract of nutmeg seeds. The confirmation of formed bionanocomposite membrane film was performed by various spectroscopic and microscopic techniques. The XRD spectrum and the SEM results revealed well dispersion of ZnONPs in the nutmeg oil/PU polymeric medium of the fabricated bionanocomposite film. The pre-synthesized bionanocomposite film was examined for immunomodulatory and antioxidant potentials. The outcomes revealed that bionanocomposite displayed strongest immunomodulatory effects on RAW 264.7 macrophages in proliferation activity, neutral red cell phagocytosis, levels of cytokines production and their mRNA expression as compared to ZnONPs and nutmeg oil. The enhanced immunomodulatory potential of bionanocomposite was highly related to existence of chemical constituents of nutmeg oil, small size, and large surface area of ZnONPs, and branching characteristics of PU. The fabricated bionanocomposite film also showed high antioxidant potential against both DPPH and ABTS radical scavenging assays. The interaction of organic and inorganic constituents with natural material has clearly activated the immunomodulatory and antioxidant properties of the bionanocomposite.

## Figures and Tables

**Table 1:** Chemical analysis of nutmeg oil by GC-MS.

S. No	Retention Time (min)	Identification	Peak Area (%)
**1**	2.23	Bicyclo[3.1.0] hex-2-ene	1.23
**2**	2.78	α-Pinene	9.16
**3**	3.86	β-Pinene	8.89
**4**	4.15	Sabinene	5.45
**5**	4.56	Myrcene	2.74
**6**	5.67	β-Phellandrene	7.89
**7**	6.26	β-Myrcene	1.08
**8**	6.52	γ-Terpinene	2.04
**9**	7.02	cymene	0.89
**10**	7.34	2-Carene	0.86
**11**	7.69	δ-Limonene	3.23
**12**	8.86	3-Carene	1.23
**13**	9.23	Camphene	1.04
**14**	10.28	2-Cyclohexene-1-ol	0.76
**15**	10.62	Linalyl butanoate	0.56
**16**	11.26	1-Methyl-4(1-methylethyl)2-cyclohexen-1-ol (cis)	1.06
**17**	12.26	3-Cyclohexene-1-ol	3.24
**18**	12.54	3-Cyclohexene-1-methanol	0.84
**19**	12.69	1-Methyl-4(1-methylethyl)2-cyclohexen-1-ol (trans)	0.56
**20**	13.42	Terpinen-4-ol	2.45
**21**	13.70	1,6-Octadien-3-ol	0.90
**22**	14.28	1,3-Benzodioxole	1.62
**23**	15.24	α-Cubebene	0.62
**24**	15.62	Copaene	1.12
**25**	16.14	1,2-Dimethoxy-4-(2-propene)	2.14
**26**	16.89	safrol	1.02
**27**	17.74	Myristicin	36.54
**28**	18.12	Asarone	0.93
**29**	18.54	Methyleugenol	0.32
**30**	18.74	Anthrone	0.98
**31**	20.72	Elemicin	3.26

**Table 2:** Hydrolytic degradation of nutmeg oil/PU/ZnONPs bionanocomposite film (90%) expressed as weight loss (%).

Time (Week)	Weight Loss % of Bionanocomposite (90%)
1	0.0
2	1.38
4	6.78
6	14.34

**Table 3:** DPPH and ABTS scavenging activity of nutmeg oil, ZnONPs, and nutmeg oil/PU/ZnONPs bionanocomposite.

Sample	DPPH Radical Scavenging Activity			ABTS Radical Scavenging Activity		
	Conc. (µg mL^−1^)	Scavenging Ability (%)	IC_50_ (µg mL^−1^)	Conc. (µg mL^−1^)	Scavenging Ability (%)	IC_50_ (µg mL^−1^)
Nutmeg oil	25 50 100	28.34 ± 0.62 31.83 ± 0.87 38.53 ± 0.22	0.57 ± 0.32	25 50 100	29.21 ± 0.14 36.63 ± 0.32 37.32 ± 0.40	0.29 ± 0.13
ZnONPs	25 50 100	53.52 ± 0.74 55.23 ± 0.52 56.18 ± 0.42	0.55 ± 0.14	25 50 100	58.18 ± 0.20 62.34 ± 0.42 64.25 ± 0.12	0.25 ± 0.15
Nutmeg oil/PU/ZnONPs bionanocomposite	25 50 100	61.32 ± 0.22 65.14 ± 0.52 66.89 ± 0.10	0.28 ± 0.22	25 50 100	64.52 ± 0.10 68.24 ± 0.56 69.57 ± 0.80	0.49 ± 0.36
Ascorbic acid	25 50 100	76.52 ± 0.12 78.28 ± 0.82 79.35 ± 0.21	0.38 ± 0.38	25 50 100	74.43 ± 0.57 76.32 ± 0.24 78.48 ± 0.76	0.52 ± 0.26

## Data Availability

The data support the findings of this study are included within the text.
